# Linking nutritional regulation of *Angptl4, Gpihbp1, and Lmf1* to lipoprotein lipase activity in rodent adipose tissue

**DOI:** 10.1186/1472-6793-12-13

**Published:** 2012-11-23

**Authors:** Olessia Kroupa, Evelina Vorrsjö, Rinke Stienstra, Frits Mattijssen, Stefan K Nilsson, Valentina Sukonina, Sander Kersten, Gunilla Olivecrona, Thomas Olivecrona

**Affiliations:** 1 Department of Medical Biosciences/Physiological Chemistry, Umeå University, Umeå, SE-90187, Sweden; 2 Nutrition, Metabolism and Genomics group, Division of Human Nutrition, Wageningen University, Wageningen, 6700EV, The Netherlands; 3 Present address: Department of Medicine, University of Gothenburg, Gothenburg, SE-405 30, Sweden

**Keywords:** Gene expression, Insulin, Gene inactivation, Cycloheximide, Actinomycin D, Transcription, Translation, Posttranslational

## Abstract

**Background:**

Lipoprotein lipase (LPL) hydrolyzes triglycerides in lipoproteins and makes fatty acids available for tissue metabolism. The activity of the enzyme is modulated in a tissue specific manner by interaction with other proteins. We have studied how feeding/fasting and some related perturbations affect the expression, in rat adipose tissue, of three such proteins, LMF1, an ER protein necessary for folding of LPL into its active dimeric form, the endogenous LPL inhibitor ANGPTL4, and GPIHBP1, that transfers LPL across the endothelium.

**Results:**

The system underwent moderate circadian oscillations, for LPL in phase with food intake, for ANGPTL4 and GPIHBP1 in the opposite direction. Studies with cycloheximide showed that whereas LPL protein turns over rapidly, ANGPTL4 protein turns over more slowly. Studies with the transcription blocker Actinomycin D showed that transcripts for ANGPTL4 and GPIHBP1, but not LMF1 or LPL, turn over rapidly. When food was withdrawn the expression of ANGPTL4 and GPIHBP1 increased rapidly, and LPL activity decreased. On re-feeding and after injection of insulin the expression of ANGPTL4 and GPIHBP1 decreased rapidly, and LPL activity increased. In ANGPTL4^−/−^ mice adipose tissue LPL activity did not show these responses. In old, obese rats that showed signs of insulin resistance, the responses of ANGPTL4 and GPIHBP1 mRNA and of LPL activity were severely blunted (at 26 weeks of age) or almost abolished (at 52 weeks of age).

**Conclusions:**

This study demonstrates directly that ANGPTL4 is necessary for rapid modulation of LPL activity in adipose tissue. ANGPTL4 message levels responded very rapidly to changes in the nutritional state. LPL activity always changed in the opposite direction. This did not happen in Angptl4^−/−^ mice. GPIHBP1 message levels also changed rapidly and in the same direction as ANGPTL4, i.e. increased on fasting when LPL activity decreased. This was unexpected because GPIHBP1 is known to stabilize LPL. The plasticity of the LPL system is severely blunted or completely lost in insulin resistant rats.

## Background

Lipoprotein lipase (LPL) is produced by parenchymal cells in some tissues (e.g. adipocytes, myocytes), secreted, and transported to the luminal side of capillaries. Here the enzyme hydrolyzes triglycerides in chylomicrons and VLDL and thereby makes fatty acids available for tissue metabolism. LPL activity is rapidly modulated by the nutritional state and plays a major role in distribution of fatty acids between tissues [1,2] .

The rapid daily modulations of LPL activity are mainly post-transcriptional [1-3]. One mechanism involves changes in the proportion of active to inactive species of the enzyme without significant changes in total LPL protein [4]. Down-regulation of adipose tissue LPL activity upon fasting requires that a gene, other than the LPL gene, is switched on [5]. This implies a protein that can transform LPL from an active to an inactive form. The fasting-induced, PPAR-responsive angiopoietin-like protein-4 (ANGPTL4) has emerged as a strong candidate for the role of such an LPL-controlling protein in adipose tissue [6,7]. *In vitro* studies show that ANGPTL4 interacts with LPL and converts active LPL dimers to inactive monomers [8,9]. Inactivation of *Angptl4* in mice is associated with low plasma triglycerides and high post-heparin LPL activity [10]. A special case is that *Angptl4*^−/−^ mice on a diet with high content of saturated fat develop severe abdominal inflammation due to excessive LPL-mediated lipid uptake in mesenteric lymph nodes [11]. Similarly, a loss of function mutation in ANGPTL4 in humans is associated with low levels of plasma triglycerides [12]. Conversely, overexpression of *Angptl*4 in mice results in low LPL activity and high plasma triglycerides [10,13,14]. Taken together these data convincingly show that ANGPTL4 is involved in modulating the activity of LPL and thereby metabolism of plasma triglycerides. It should be noted however that ANGPTL4 also has effects on angiogenesis and several other processes, and that there are additional members in the ANGPTL protein family that have effects on plasma lipid metabolism [15] .

Another protein important for LPL action is the newly discovered glycosylphosphatidylinositol-anchored high density lipoprotein-binding protein 1, GPIHBP1 [16]. This protein, which is expressed only in endothelial cells, is crucial for transfer of LPL to the luminal side of the capillary endothelium [17]. Inactivation of the gene for GPIHBP1 in mice results in gross chylomicronemia. Genetic deficiency in humans produces a clinical phenotype similar to that in LPL deficiency. It is not known if GPIHBP1 has any direct role in the modulation of LPL action. Like *Angptl4*[6] the *Gpihbp1* is PPAR-responsive [18], suggesting that the two genes might be similarly regulated.

A third, newly discovered, protein involved with the lipase system is the lipase maturation factor (LMF1). This is an ER-based chaperone which appears to be necessary for maturation of LPL and the related hepatic lipase and endothelial lipase into their active forms [19,20].

In this paper we have explored how this landscape of LPL controlling proteins behaves in rat adipose tissue under a number of conditions previously shown to be associated with rapid changes of LPL activity. Specifically we have studied (1) if ANGPTL4 is needed for the rapid modulation of LPL activity; (2) if the message levels show circadian oscillations, (3) at what rates the messages for the three LPL-controlling proteins and the proteins themselves are being turned over, (4) how the messages for the LPL controlling proteins change on changes in the nutritional state, known to cause large changes of adipose tissue LPL activity and (5) what happens to the expression of the three proteins when rats become insulin resistant.

## Results

### Circadian rhythm

**Figure 1:**
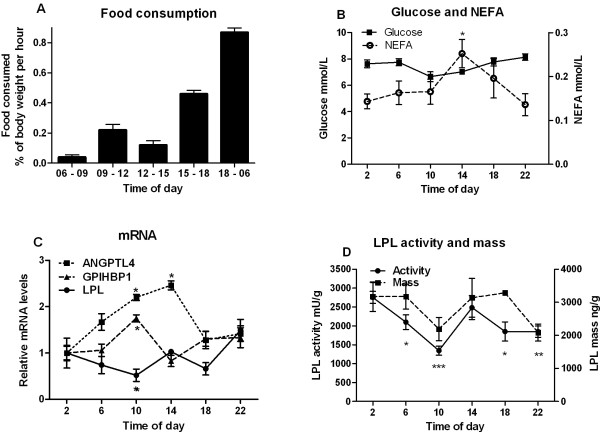
**Circadian changes.** Groups of young rats (n=6) fed *ad lib* were sacrificed at the indicated times over a 24 hour period. Epididymal adipose tissue and blood were taken for analyses. (**A**) Food consumption was measured in a separate group of rats over a 3-day period. These data are expressed as % of body weight per hour. (**B**) Glucose concentration in blood (■) and NEFA concentration in plasma (s○). (**C**) mRNA abundance for LPL (●), ANGPTL4 (■) and GPIHBP1 (▲) in epididymal adipose tissue. (**D**) LPL activity (●) and mass (■) in the adipose tissue. Statistical comparisons were made against the values at 2:00 h.

The expressions in adipose tissue of many of the genes involved in energy metabolism undergo circadian changes [21]. We followed the changes with time of day of a number of parameters related to LPL activity (Figure 1). Rats eat mainly during the dark period (18:00 h – 6:00 h). Mean food consumption was 0.88 ± 0.10% of body weight per hour during the night (Figure 1A). During the first hours of the light period (6:00 h – 9:00 h) the rats ate 0.04 ± 0.01% of body weight per hour. This is less than 5% of what they ate per hour during the night. From 9:00 h −15:00 h food consumption per hour was about one quarter of that during the dark period. During the last hours of the light period, 15:00 h – 18:00 h, food consumption increased to about half of that during the dark period.

The changes in plasma glucose and non-esterified fatty acids (NEFA) over the day were moderate (Figure 1B). Adipose tissue LPL mRNA (Figure 1C) showed its highest value at 22:00 h. This was 2.8 fold higher than the lowest value (at 10:00 h, p=0.028). Changes in GPIHBP1 mRNA (Figure 1C) were modest and the tendency was opposite to that for LPL mRNA, with higher values during the light than during the dark period. The increase in ANGPTL4 mRNA during the light period was more pronounced compared to GPIHBP1, but the levels were down again by 18.00 h. LPL activity (Figure 1D) in adipose tissue showed its highest value at 2:00 h and its lowest value at 10:00 h (p<0.01). The difference was about two-fold. LPL mass (Figure 1D) followed a similar pattern, and the ratio between LPL activity and mass (specific activity) did not change significantly.

### Response of the proteins studied and of LPL activity when mRNA or protein synthesis was blocked

**Figure 2:**
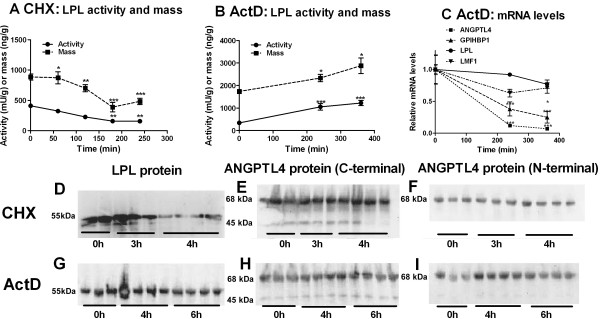
**Time courses for the effects of cycloheximide and actinomycin D.** Young rats were fasted overnight and were then injected with cycloheximide (CHX, panels **A** and **D**-**F**) or ActD (panels **B**, **C** and **G**-***I***) starting at 8:00 h. Groups of rats (n=5) were sacrificed at the indicated times over a 4 or 6 hour period. Rats for the time 0 groups were not injected and were sacrificed within one hour from the start of the experiment. Epididymal adipose tissue was taken for analyses. (**A**) LPL activity (●) and mass (■) after CHX. (**B**) LPL activity (●) and mass (■) after ActD (**C**) mRNA levels for ANGPTL4 (■), GPIHBP1 (▲), LPL (●) and LMF1 (▼). mRNA was calculated relative to 18S mRNA. The value at time 0 was set to 1 and values at following time points calculated relative to this. (**D**-**I**) Western blots of adipose tissue as labeled in the figure. The antibodies to C-terminal ANGPTL4 used in panels E and H recognize both full-length ANGPTL4 and the fibrinogen-like C-terminal domain. The antibodies to the N-terminal domain of ANGPTL4, used in panels **F** and **I**, identifies the full-length protein but do not see the C-terminal domain.

To directly study the turnover of the proteins involved, cycloheximide was injected in rats to block synthesis of new proteins (Figure 2). LPL activity decreased by 60% in 3 hours (Figure 2A). LPL mass, measured by ELISA (Figure 2A), and estimated from Western blots (Figure 2D), decreased at a similar rate as the activity. These data are in line with earlier observations that the LPL protein turns over rapidly [22,23]. In contrast, ANGPTL4 protein showed bands of similar intensity on western blots throughout the four hours studied (Figure 2E and F). The pattern was the same when antibodies to the C-terminal (Figure 2E) or N-terminal domain (Figure 2F) of ANGPTL4 were used. Almost all of the protein was full length (here 68 kDa compared to MW standards). With antibodies to the C-terminal domain small amounts of a 45 kDa fragment was seen (Figure 2E), probably representing the fibrinogen-like domain of ANGPTL4. These data show that the ANGPTL4 protein turns over relatively slowly, much slower than the LPL protein. The turnover of the GPIHBP1 and LMF1 proteins could not be studied since suitable antibodies were not available.

To directly study the turnover of the corresponding messages, the transcription blocker actinomycin D (ActD) was injected (Figure 2). Fasted rats were used since the level of ANGPTL4 and GPIHBP1 messages in adipose tissue are several-fold higher in the fasted than in the fed state [6,18]. In concert with earlier studies [5], LPL activity in adipose tissue rose from 340 ± 43 to 1225 ± 112 mU/g in six hours after ActD (Figure 2B). The value for LPL mRNA decreased by 8% by 4 h and by 33% by 6 h (Figure 2C), but these changes did not reach statistical significance. LPL mass as measured by the ELISA increased by 40% (p<0.05) in 6 h. Consistent with these data, Western blots showed that the level of LPL protein was essentially stable over the six h studied (Figure 2G). These data confirm that the LPL transcript is relatively stable.

While LPL activity went up, mRNA levels for ANGPTL4, GPIHBP1 and LMF1 were reduced by 93%, 75% and 29% by six hours after ActD (Figure 2C). These results show that the ANGPTL4 and GPIHBP1 transcripts are turned over rapidly. On Western blots, the ANGPTL4 protein showed bands of similar intensity throughout the six hours (Figure 2H and I). This pattern was the same when antibodies to the C-terminal or N-terminal domain were used. Almost all of the protein was full length but traces of a 45 kDa fragment were seen with the antibody specific for the C-terminal domain of ANGPTL4. These data again show that the ANGPTL4 protein turns over relatively slowly.

### Early response to food withdrawal

**Figure 3:**
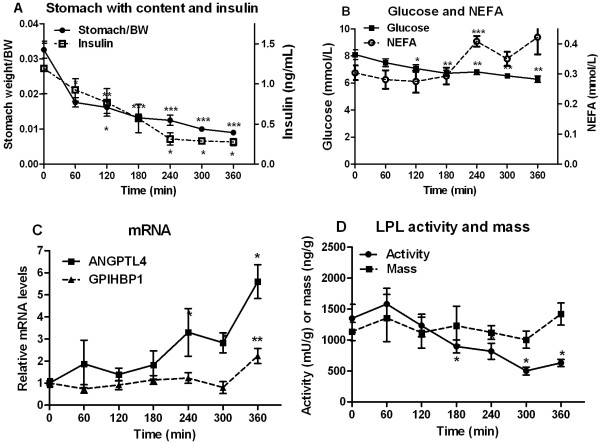
**Response to food deprivation.** Food was withdrawn from young rats at 8:00 h. Animals were then sacrificed at the indicated time points over a six hour period (n=6 at each time). The experiment was conducted over three days. Rats for the different time points were randomly included on each day. Epididymal adipose tissue and blood were taken for analyses and the stomach with its content was cut out and weighed. (**A**) Weight of the stomach expressed as fraction of body weight (●) and concentration of insulin in plasma (□) (**B**) Concentrations of glucose (■) and NEFA (○) in blood and plasma, respectively (**C**) mRNA abundance for ANGPTL4 (■) and GPIHBP1 (▲). mRNA was calculated relative to 18S mRNA. The value at time 0 was set to 1 and values at following time points were calculated relative to this. (**D**) LPL activity (●) and mass (■).

To study the early events in adaptation of the LPL system in adipose tissue to fasting, food was withdrawn from rats in the morning. At this time the animals ate rather little (Figure 1A). Their stomachs were about half full and emptied almost completely within two hours after food was removed. Insulin (Figure 3A) and glucose (Figure 3B) decreased whereas NEFA increased (Figure 3B). Triglycerides decreased, presumably because the inflow of chylomicrons from the intestine faded (data not shown). LPL mRNA in adipose tissue did not change significantly, in accordance with earlier observations [24] (data not shown). The level of mRNA for LMF1 also did not change significantly (data not shown). In contrast, the levels of ANGPTL4 mRNA and GPIHBP1 mRNA increased markedly (about 6-fold and 2-fold, respectively) in adipose tissue over the six hours studied (Figure 3C). LPL activity in adipose tissue had decreased by 40% at four hours and by 53% at six hours (Figure 3D). In contrast there was no significant change of LPL mass (Figure 3D). All of these responses to food withdrawal were similar in a parallel experiment on female rats, but data are shown only for males. These data indicate that short term fasting is associated with reciprocal changes in adipose ANGPTL4 mRNA and LPL activity.

### Relation to changes in expression of other genes

To get a perspective on the rapid and large response of *Angptl4* transcript on fasting [8] we carried out an array analysis of the changes of RNA abundances in adipose tissue under the conditions used for our experiments. For this, food was removed from one group of rats in the early morning. Seven h later the rats were sacrificed, epididymal adipose tissue was cut out, RNA prepared and analyzed on Illumina arrays. N = 6 for both the fasted and the *ad lib* group. After filtering the data for P < 0.01 and signal intensity > 50, there remained 22 transcripts that had increased, and 21 that had decreased by a factor of 2.0 or more.

Sorting by foldchange+, *Angptl4* came out as number four from the top *(*foldchange 3.37, p < E _*_ 10^-17^*)*, preceded only by *Pdk4*, pyruvate dehydrogenase kinase 4, a key regulator of glycolysis/glyceroneogenesis, *Gpr109a*, the ketone body/niacin receptor, a key regulator of lipolysis, and *RGD1565690*_predicted, a gene of unknown function (Table 1). In concert with the results of the experiment in Figure 3 and earlier observations [24], the expression of *LPL* did not change significantly (foldchange 1.02). Neither *Gpihbp1* or *LmfF1* was represented on this chip.

**Table 1:** The eight genes that increased their expression in adipose tissue the most when food was removed

**Symbol**	**Foldchange +**	**DiffScore**
Pdk4	4.97	70.6
Gpr109a	3.86	54.8
RGD1565690_predicted	3.83	55.7
Angptl4	3.58	330.8
Per1	3.25	39.4
Pfkfb3	3.15	12.7
Pck1	2.91	40.9
Net1	2.82	56.3
Lpl	1.02	0.00004

Food was removed from one group of rats in the early morning. Seven h later the rats were sacrificed, epididymal adipose tissue was cut out, RNA prepared and analyzed on Illumina arrays. N = 6 for both the fasted and the *ad lib* group. Full data are deposited at NCBI GEO, accession no GSE 41800.

### Responses to re-feeding

**Figure 4:**
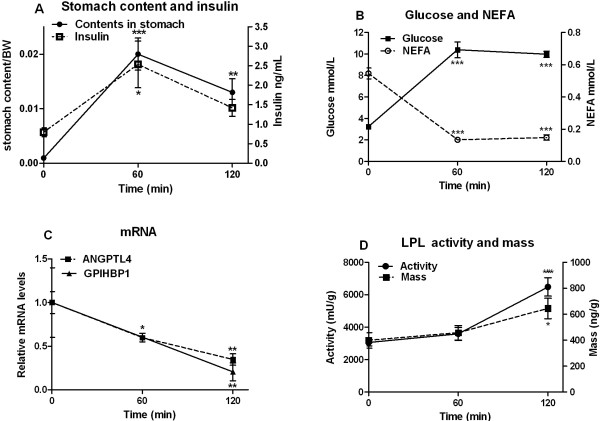
**Responses to re-feeding.** Young rats were fasted overnight (16 h, from 16:00 h). Food was given back (at 8:00) and groups of rats (n=5) were sacrificed after one or two hours. Time = 0 min were a group of rats sacrificed within one h of continued fasting. Epididymal adipose tissue and blood was collected and analyzed. The stomach was cut out, weighed, opened and rinsed and then weighed again. The difference is the “contents in stomach” and is expressed as fraction of body weight (**A**) Weight of contents in stomach (●) and concentration of insulin in plasma (□) (**B**) Concentrations of glucose (■) and NEFA (○) in blood and plasma, respectively. (**C**) mRNA abundance for ANGPTL4 (■) and GPIHBP1 (▲). mRNA was calculated relative to 18S mRNA. The value at time 0 was set to 1 and values at following time points were calculated relative to this. (**D**) LPL activity (●) and mass (■).

To study a situation when adipose tissue LPL activity increases rapidly we turned to re-feeding as a physiological model that we had used before [8]. For this, rats were fasted overnight and food was given back in the morning. The animals started to eat within 5 to 10 min. By one hour their stomachs contained 2.0 ± 0.03 g/100 g body weight (Figure 4A), similar to what was found in the stomach of regularly fed rats (Figure 3A). Insulin (Figure 4A) followed a time course similar to that for weight of stomach contents. Glucose increased (Figure 4B), and NEFA decreased (Figure 4B). ANGPTL4 and GPIHBP1 mRNA levels decreased rapidly and after two hours were only 35 and 21% of the initial levels, respectively (Figure 4C). LPL activity and mass increased (Figure 4D). Thus, similar to fasting, fasting-refeeding is associated with reciprocal changes in adipose ANGPTL4 and GPIHBP1 mRNA on the one hand, and LPL activity on the other hand.

### Response of the LPL system to fasting and re-feeding in Angptl4 ^−/−^ mice

**Figure 5:**
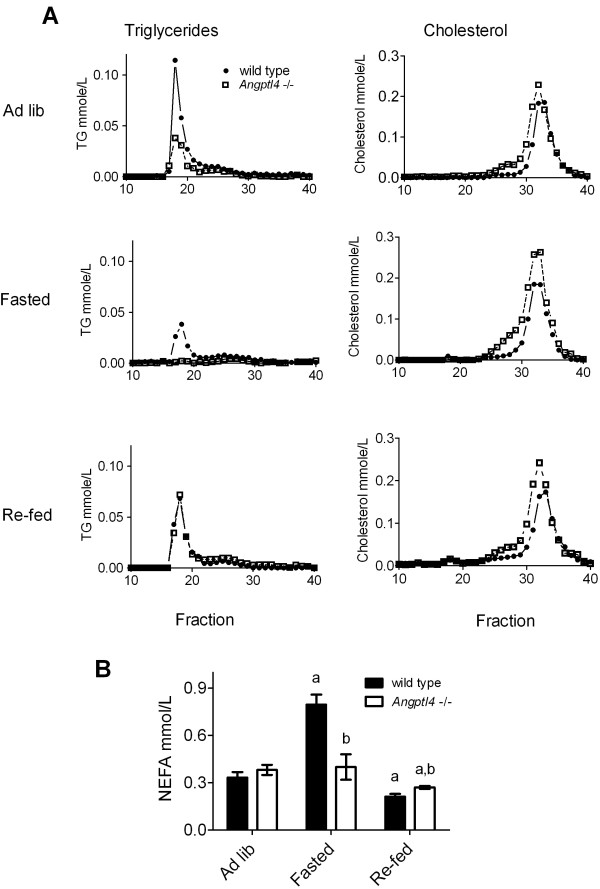
**Plasma lipids in Angptl**^**−/−**^**mice.** Wild-type and *Angptl*^*−/−*^ mice were fasted overnight or fed *ad lib*. Food was given back to groups of fasted mice at 6:00 h (re-fed), while others continued fasting or eating (n=6 per group). All animals were sacrificed between 9:00 h – 10:00 h. (**A**) FPLC analyses of pooled plasma samples representing the 6 different groups. (**B**) Fatty acids (NEFA) in individual plasma samples. Statistically significant (p<0.05) differences are indicated by: a when comparing fasted or re-fed animals to *ad lib* for the same genotype b when comparing the two genotypes for the same nutritional state.

To directly investigate the role of ANGPTL4 in the rapid modulation of LPL activity we turned to studies in mice in which *Angptl4* had been inactivated [10]. Groups of mice (n=6) were fed *ad lib,* fasted over night, or fasted over night and then re-fed in the morning for 3 h. Plasma lipid levels were in accordance with previous results on *Angptl4*^−/−^ mice [10]. Separation of lipoprotein classes by FPLC high-lighted that the fasted *Angptl4*^*−/−*^ mice had no TG in the VLDL fraction, while after re-feeding the levels of VLDL TG were similar in *Angpt4*^−/−^ compared to wild-type mice (Figure 5A). The peak of HDL cholesterol was slightly higher in *Angptl4*^*−/−*^ and was shifted towards larger particles in all three groups of mice compared to wild-type (Figure 5A). The increase in NEFA on fasting was blunted in the *Angptl4*^*−/−*^ mice (Figure 5B). This is in accordance with a role of ANGPTL4 for intracellular lipolysis [25].

**Figure 6:**
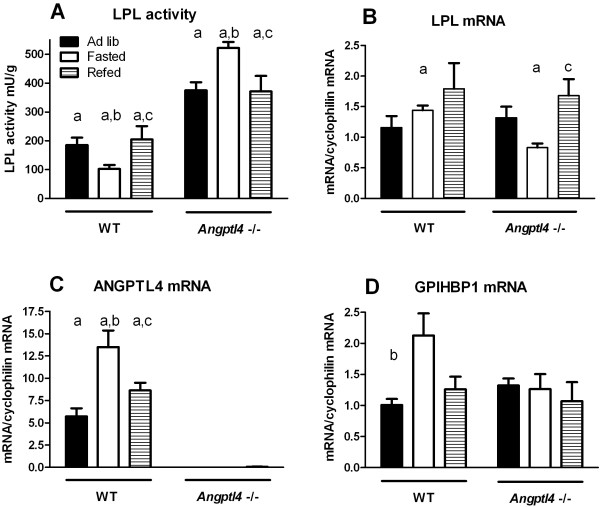
**Response of the LPL system to fasting and re-feeding in Angptl4**^**−/−**^**mice*.*** Epididymal adipose tissue from the same mice as described in Figure 5 was cut out and analyzed for LPL activity and mRNA abundance for LPL, ANGPTL4, and GPIHBP1. The levels of mRNA were calculated relative to cyclophilin mRNA. Statistically significant (p < 0.05) differences are indicated by: a when comparing *Angptl4*^−/−^ and WT for the same nutritional state b when comparing fasted and *ad lib* for the same genotype c when comparing refed and *ad lib* for the same genotype.

In the wild-type animals, fasting caused a decrease of adipose tissue LPL activity, as expected (Figure 6A). In contrast, fasting caused an increase of adipose tissue LPL activity in the *Angptl4*^−/−^ mice compared to fed or re-fed *Angptl4*^−/−^ animals. LPL activity was significantly higher in all three nutritional states in *Angptl4*^−/−^ compared to wild-type animals, indicating that ANGPTL4 represses LPL activity to some extent also in fed animals. ANGPTL4 mRNA was about two-fold increased in fasted compared to *ad lib* fed wild-type mice, while, as expected, there was no detectable ANGPTL4 mRNA in the *Angptl4*^−/−^ mice (Figure 6C). GPIHBP1 mRNA was also increased about two-fold in fasted wild-type mice compared to the fed groups. Interestingly, this difference was not seen in the *Angptl4*^−/−^ mice (Figure 6D). LPL mRNA was not significantly changed in wild-type mice, but was decreased in the fasted *Angptl4*^−/−^ mice compared to the fed groups (Figure 6B). Taken together these data demonstrate that the reduction in adipose LPL activity upon fasting is dependent on increased expression of ANGPTL4.

### Response to insulin

**Figure 7:**
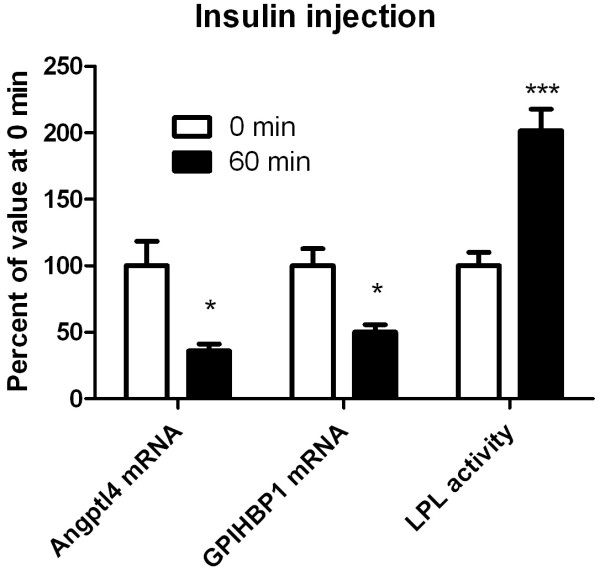
**Effects of insulin on expression of LPL activity, ANGPTL4 and GPIHBP1.** Insulin was injected i.p. to young rats fasted for 6 hours (from 8:00 h, n=6 per group). The rats were sacrificed 60 min after injection. Time = 0 min were non-injected rats sacrificed before the injected rats. LPL activity and mass and mRNA abundance for ANGPTL4 and GPIHBP1 were measured in epididymal adipose tissue.

To study direct effects of insulin on the LPL system, fasted rats were given an intraperitoneal injection of insulin, 1 U/kg body weight, which is in the range used for insulin tolerance tests in rodents [26]. The injection resulted in levels of insulin in blood that were about five times higher than the mean level in controls at 60 min. Glucose and NEFA both decreased (data not shown). Already one hour after the injection, adipose tissue LPL activity had almost doubled (Figure 7). LPL mass did not change significantly (data not shown). ANGPTL4 and GPIHBP1 mRNA both decreased (Figure 7). One hour after injection of insulin the levels were 33% and 50% of the initial levels, respectively.

### Changes with age and/or weight of the rats

Previous studies have shown that the response of adipose tissue LPL activity to feeding/fasting is blunted in old, obese rats [5,27]. To probe this we studied rats at three different ages, 5 weeks old, lean, body weight 199 ± 20 g; 25 weeks old, moderately obese, body weight 695 ± 66 g; and 52 weeks old, grossly obese, body weight 1006 ± 82 g. One set of rats from each age group was fasted overnight and compared to *ad lib* fed controls.

**Figure 8:**
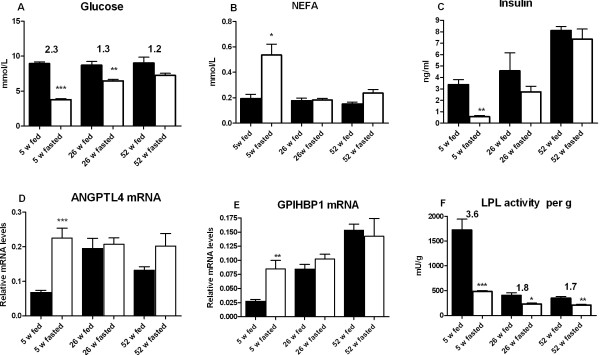
**Effects of age and/or obesity.** Groups of rats of different ages (5 weeks, 26 weeks and 52 weeks) were either fed *ad lib* (solid bars) or fasted overnight for 16 h (open bars). At 8:00 h groups of fed and fasted rats of the indicated age were sacrificed (n=5 per group). The epididymal fat body was cut out and weighed. Blood was also taken for analyses of (**A**) glucose, (**B**) NEFA and (**C**) Insulin. mRNA levels for ANGPTL4 (**D**) and GPIHBP1 (**E**) and are expressed relative to 18S RNA. (**F**) LPL activity expressed per g wet weight of tissue. Statistical comparisons are made between fed and fasted animals. The numbers above some of the bars represent the relative change from the fasted to the fed state.

In the fasted state, the young lean rats had low blood glucose, about 3.3 mmol/L. The intermediate and old rats had much higher blood glucose, about 6.0 mmol/L (Figure 8A). In the fed state the three groups had similar blood glucose levels, around 8 mmol/L. The fed/fasted ratio for glucose was around 2.3 in the young, lean rats, but only 1.3 and 1.2 in the old and intermediate groups, respectively. NEFA levels were similar in all three groups in the fed state (Figure 8B). On fasting, the level increased almost three-fold in the young rats, but did not change significantly in the intermediate or old rats. Plasma insulin levels (Figure 8C) showed large differences between the groups. The fasting level was below 0.6 ng/ml in the young rats, four times higher in the intermediate group and 12 times higher in the oldest group. The fed/fasted ratio was about 5 in the young lean rats, but only 1.35 in the intermediate group. In the old group insulin levels in plasma did not differ between fed or fasted animals.

In accordance with the observations for food withdrawal (Figure 3) and re-feeding (Figure 4), ANGPTL4 and GPIHBP1 mRNA levels were much higher in the fasted than in the fed state in the young rats (Figure 8D and E). In contrast there were no statistically significant changes with nutritional state in the older rats.

Adipose tissue LPL activity, expressed per gram wet tissue weight, was high in the young rats, about 1600 mU/g. In the intermediate and old rats the activity was less than one-third (Figure 8F). These rats had much larger fat pads than the young lean ones so that the total LPL activity per pad was actually larger in the older groups (2.1-fold and 3.6-fold for the intermediate and old rats, respectively), compared to the young rats. Remarkably, the response to nutritional state was more pronounced in the young rats with a fed/fasted ratio more than twice as high as that in the two older groups (Figure 8F).

## Discussion

The present study reinforces previous observations that rapid modulation of LPL activity (in rodent adipose tissue) is not exerted at the level of LPL gene expression [1,24,27,28]. LPL mRNA levels remained essentially stable under the conditions we tested. Likewise, the mRNA for LMF1, an ER protein needed for proper maturation of LPL into its active form [19,20], did not change significantly, in accordance with an earlier study in Zucker diabetic rats [29]. In contrast, the mRNAs for ANGPTL4 and GPIHBP1, changed much more rapidly than most mRNAs in mammalian cells [30]. These two proteins interact with LPL and we will designate them collectively as ‘LPL controlling proteins’. There may well be more, yet undiscovered, proteins that participate in the LPL system. A major mechanism for the modulation of adipose LPL activity is conversion of catalytically active LPL dimers into inactive monomers [4,8]. This process is virtually irreversible [31,32]. The LPL protein, active or inactive, turns over with a half-life of less than two hours [22,23] which is much more rapid than the turnover of most proteins in mammalian cells [30]. Hence, the overall design of the LPL system appears to be relatively constant production and secretion of short-lived enzyme molecules that either retain or loose their catalytic activity in response to a number of controlling proteins. This design may have evolved to meet the need to modulate the activity of secreted/extracellular LPL molecules.

The expression of many genes involved in energy metabolism undergo profound circadian oscillations in the adipose tissue presumably to adapt the animal to predictable changes in the environment [21], LPL mRNA, mass and activity all shoved higher values in the middle of the dark period. For LPL activity the amplitude was about two-fold, in accordance with previous studies [24,33-37]. ANGPTL4 and GPIHBP1 mRNA showed modest changes opposite in direction to that for LPL. Hence, it appears that the LPL system displays a moderate circadian oscillation in phase with the eating behavior, but can respond rapidly and profoundly whenever food becomes scarce. Of note, the circadian oscillations of adipose tissue LPL are more pronounced in mice, with a more than three-fold higher activity in the middle of the dark period compared to the middle of the light period [38]. A significant circadian variation has also been reported for post-heparin LPL activity in humans [39].

A possible confounding factor for studies of how LPL activity is modulated in adipose tissue is changes in the rate at which proteins are being synthesized in the adipocytes [1]. Parkin et al. found that insulin more than doubled the rate of incorporation of amino acids into proteins in rat fat pads and caused a corresponding increase in LPL activity [40]. Other groups have reported similar observations [1,2]. Kern and his associates have described a more specific mechanism that affects LPL synthesis whereby stimulation of the protein kinase A system leads to formation of a protein complex that binds to the 3’UTR of LPL mRNA and blocks its translation [28]. It seems likely that part of the early responses of LPL activity in our studies, e.g. after injection of a large dose of insulin, reflect decreased/increased rates of LPL translation. This can not, however, fully explain the changes seen. For instance, during food deprivation (Figure 3) LPL activity decreased by more than 50%, while there was no significant change in LPL mass.

The experiment on *Angptl4*^−/−^ mice clearly demonstrated the important role of ANGPTL4 for suppression of adipose tissue LPL activity in the fasted state. Even though LPL mRNA levels were significantly reduced in adipose tissue of the *Angptl4*^−/−^ mice, compared to all other groups of mice, LPL activity was the highest in adipose tissue of fasted *Angptl4*^−/−^ mice. Also in the *ad lib* fed and the re-fed states, adipose tissue LPL activity was significantly higher in *Angptl4*^−/−^ mice than in wild-type mice, indicating that some LPL is depressed by ANGPTL4 even under conditions when LPL activity is at demand. These data are compatible with those of Köster et al. [10], demonstrating 2–3 fold higher LPL activity in post-heparin plasma in *Angptl4*^−/−^ mice compared to wild-type mice in both fed and fasted animals. Similarly, blocking transcription by ActD led to a decrease in ANGPTL4 mRNA levels in adipose tissue and to a 3-fold increase in LPL activity in fasted rats [8], and to a 3-fold increase in post-heparin plasma LPL activity in *ad lib* fed rats [41]. In fed animals, most of the LPL activity in post-heparin plasma originates from adipose tissue, while in fasted animals the dominating source is presumably skeletal muscle and heart [38,42]. Taken together the data demonstrate that ANGPTL4 is an important modulator of LPL activity in both fasted and fed animals.

The present data show that ANGPTL4 mRNA turns over rapidly in adipose tissue, in concert with an earlier study [8]. When transcription was blocked by injection of ActD, the mRNA decreased more than 90% in 4 hours. The mRNA level responded rapidly to the perturbations of the nutritional state that we used. It increased more than 200% from 3 to 6 hours after food deprivation. It decreased by about 50% in one hour after injection of insulin and by about 65% within two hours after re-feeding of rats that had fasted overnight. In all of these situations, the expression of *Angptl4* changed as expected for a gene that negatively controls LPL activity. Whenever the expression of *Angptl4* increased, LPL activity decreased and conversely when the expression of *Angptl4* decreased, LPL activity increased. The time courses for the changes were compatible with a major role for ANGPTL4 in modulation of LPL activity, if one takes into account that the general rate of protein synthesis, and hence LPL synthesis, probably changed (see above). In contrast to the rapid changes of ANGPTL4 message levels, the ANGPTL4 protein remained essentially unchanged over several hours, as evaluated by Western blots. This was true whether we used antibodies to the N-terminal or C-terminal domains, and was true both in experiments where syntheses of new protein was blocked by cycloheximide and in experiments where message levels changed several-fold in response to a transcription block or in response to food withdrawal, re-feeding or insulin injection. This suggests compartmentalization, such that only newly synthesized ANGPTL4 protein can inactivate LPL. Within cells the ANGPTL4 protein exists as monomers but they form oligomers when they reach the cell surface [43]. It is only after oligomerization that the ANGPTL4 protein can interact with and inactivate LPL [12,43]. Hence, it is possible that LPL and ANGPTL4 monomers do not interact when they travel through the secretory pathway, but there is a critical event when the proteins emerge at the cell surface that triggers oligomerization of ANGPTL4 and thereby inactivation of LPL. LMF1 may have a role here [20]. Once LPL transfers to the endothelial cells the enzyme may be rescued by interaction with heparan sulfate proteoglycans [44] and/or GPIHBP1 [45].

The message for GPIHBP1 also turned over rapidly. When transcription was blocked by ActD the message decreased by more than 60% within 4 hours. It increased 4-fold from 3 to 6 hours after food deprivation. It decreased by about 50% in one hour after injection of insulin and by about 80% within 2 hours after re-feeding of overnight fasted rats. This is in accordance with the studies of Davis et al. who found that GPIHBP1 message in adipose tissue is higher in 16 h fasted than in fed rats [18]. Comparing the amplitudes of the changes, *Gpihbp1* was at least as responsive as *Angptl4*. This is impressive considering that array analysis showed that *Angptl4* was one of the genes whose expression had increased most seven hours after food withdrawal (Table 1). The changes of GPIHBP1 mRNA were in the same direction as the changes of ANGPTL4 mRNA in all the situations that we studied. This is surprising. According to present hypotheses ANGPTL4 protein suppresses LPL activity. GPIHBP1 on the other hand stabilizes the enzyme and promotes its delivery to the site of action at the vascular endothelium [45]. One should note that the changes presumably take place in different cells in the tissue. *Gpihbp1 i*s expressed in endothelial cells [16] whereas *Angptl4* is presumably expressed mainly in adipocytes. It is possible that GPIHBP1 not only delivers LPL to the luminal side of the endothelium, but that under certain circumstances, like during fasting, GPIHBP1 may predominantly transport LPL in the opposite direction leading to LPL-inactivation and or degradation within the tissue [46].

Rapid, tissue-specific modulation of LPL activity is important for whole body energy homeostasis by directing lipid uptake to the appropriate tissues and limiting the need for re-transport [1,2]. Earlier studies have shown that the response of adipose tissue LPL activity to feeding-fasting becomes blunted as rats grow older and become obese [5,24]. The present study confirms these observations and links them to the development of insulin resistance. In young, lean rats (5 weeks old) adipose tissue LPL activity decreased by a factor of 3.6 – 3.9 on fasting overnight. In older, obese rats the response was less than half, 1.7 – 1.9-fold. The older rats appeared to be insulin-resistant. Fasting blood glucose and insulin was elevated compared to the young rats and the difference fed versus fasted was less for all parameters studied. It is of interest to note that these rats were not manipulated in any way but housed by normal routines and fed chow *ad lib*[47]*.* The responses of the LPL controlling genes, *Angptl4* and *Gpihbp1* were blunted. The large change in GPIHBP1 mRNA (about 3-fold) seen when young rats were fasted was completely abolished in the older rats. For ANGPTL4 mRNA some response remained but it was much less than in the young rats. It is of interest to note that the values seen in either the fed or fasted state in the older rats were similar to those seen in fasted young rats. Hence, it appears that it was the ability to down-regulate the expression in the fed state that caused the loss of metabolic plasticity. In line with this it was recently shown that mRNA of Angptl4 is upregulated in diabetic mice [48] whereas insulin inhibited Angptl4 mRNA expression in 3 T3-L1 adipocytes [49]. Moreover, FFA, which are increased in the insulin-resistant state, were shown to upregulate mRNA expression of Angptl4 in human adipocytes [50]. A study on groups of human subjects (young, lean compared to old, obese with or without diabetes type II) demonstrated a blunting of the response of *Angptl4* to feeding/fasting in both groups of elderly individuals compared to the young, while no effect was in this case seen on *GPIHBP1* expression [42]. Bergö et al. found that blocking transcription by ActD (which relieves the suppression of LPL activity) increased adipose tissue LPL activity several-fold in fasted young rats, but had only a small effect in old, obese rats [5]. These data are compatible with the hypothesis that expressions of *Angptl4* and/or *Gpihbp1* are main determinants for LPL activity in adipose tissue of rats.

Modulation of LPL activity is important for partitioning of lipids between tissues in accordance with changes in the metabolic situation [1,2]. It is becoming evident that that modulation of LPL action occurs by interplay of several factors. The central player, the LPL enzyme, appears to be produced at a relatively constant rate. The activity and the distribution of the enzyme between the endothelial cell surface and other places in the tissue are determined by LMF1, ANGPTLl4 and GPIHBP1 and perhaps other proteins in a context dependent manner. In addition LPL action is modulated by factors pertaining to the lipoprotein substrate [1]. Apolipoprotein CII is a necessary cofactor. Apolipoprotein CIII and other apolipoproteins can suppress lipase action. In this case the action of the enzyme is inhibited but not irreversibly lost.

## Conclusions

The main conclusion from this study is that ANGPTL4 is necessary for the rapid modulation of LPL activity in adipose tissue. ANGPTL4 message levels responded very rapidly to changes in the nutritional state. LPL activity always changed in the opposite direction. This did not happen in Angptl4^−/−^ mice. GPIHBP1 message levels also changed rapidly and in the same direction as ANGPTL4, i.e. increased on fasting when LPL activity decreased. This was unexpected because GPIHBP1 is known to stabilize LPL.

In old, obese rats that showed signs of insulin resistance, the responses of ANGPTL4 and GPIHBP1 mRNA and of LPL activity were severely blunted (at 26 weeks of age) or almost abolished (at 52 weeks of age). Hence, the plasticity of the LPL system is severely blunted or completely lost in insulin resistant rats.

## Methods

### Animal procedures

Inbred male Sprague–Dawley rats weighing 150–210 g were used, except in the exp on the effect of ageing/insulin resistance in Figure 8. The rats were housed with free access to a standard pellet diet and water in a 12-h light cycle (6:00 h – 18:00 h). Experiments were, unless otherwise stated, carried out with rats fed or fasted overnight (food removed at 16:00 h) or fasted for 6 h (food removed at 8:00 h). During fasting the rats were kept in cages with a perforated floor to prevent coprophagia. In experiments with re-feeding, rats were fasted overnight and then food was given back at 8:00 h. The experiment then continued for 2 h. The rats were killed by decapitation. The fat depot studied was the epididymal. Tissues were collected into Eppendorf tubes and immediately frozen in liquid nitrogen. Blood samples to be analyzed for LPL activity and metabolites were collected in EDTA blood collection tubes (Sarstedt). Plasma was obtained by centrifugation of the blood at 4°C and the samples were then stored at −70°C. the bars represent the relative change from the fasted to the fed state

Male pure-bred WT and *Angptl*4−/− mice on a C57Bl/6 background between ages 4–6 months were used [10]. Fasted mice were fasted from 15:00 h and sacrificed the next day between 9:00 h and 10:00 h. Refed mice were fasted from 15:00 h, refed with chow the next day at 6:00 h and sacrificed between 9:00 h and 10:00 h. Mice were anaesthetized with a mixture of isoflurane (1.5%), nitrous oxide (70%) and oxygen (30%). Blood was collected by orbital puncture into EDTA tubes. Mice were killed by cervical dislocation, after which tissues were excised and directly frozen in liquid nitrogen.

All animal procedures were approved by the local animal ethics committees in Umeå (rats) and Wageningen (mice), respectively.

In experiments on rats, insulin (Actrapid, Novo Nordisk A/S, Denmark) was injected intraperitoneally (1U/kg bw) to rats fasted for 6 h. Controls were injected with saline only. Actinomycin D (Sigma Aldrich), dissolved in pure ethanol to a concentration of 2 mg/ml, was injected intraperitoneally (2 mg/kg bw) to rats fasted for 6 h. Controls were injected with ethanol. Cycloheximide (Sigma Aldrich), dissolved in saline to a concentration of 35 mg/ml, was injected intraperitoneally (35 mg/kg bw) to rats fasted for 16 h. Controls were injected with saline.

### LPL activity assay

Frozen tissues were homogenized in 9 volumes of buffer at pH 8.2 containing 0.025 M ammonia, 1% Triton X-100, 0.1% SDS and protease inhibitor cocktail tablets (Complete Mini, Roche Diagnosis, Germany) using a Polytron PT 3000 Homogenizer (Kinematica). The homogenates were centrifuged for 15 min at 10,000 rpm, 4°C. Aliquots of the supernatants were used for determination of LPL activity as previously described [5] using a phospholipid-stabilized emulsion of soy bean triacylglycerols and ^3^H-oleic acid-labeled triolein with the same composition as Intralipid 10% (Fresenius Kabi, Uppsala, Sweden). The incubation was at 25°C for 100 or 120 min. One milliunit of enzyme activity corresponds to 1 nmol of fatty acids released per min. Enzyme activity is expressed per g wet tissue weight. In the exp in Figure 8 LPL activity per whole epididymal fat pad was also calculated. All samples were assayed in triplicates.

### LPL mass determination

Mass was measured by an Elisa method previously described [4,5]. Affinity purified immunoglobulins (IgY) from chicken, raised against bovine LPL were used for capture of the antigen during an overnight incubation. Bound LPL was detected with 5D2 monoclonal antibody raised against bovine LPL (a generous gift from Prof. J. Brunzell, University of Washington, Seattle WA), followed by detection with peroxidise-labelled anti-mouse IgG. LPL purified from bovine milk was used as standard.

### Quantitative RT-PCR analyses

For determination of the levels of LPL, ANGPTL4, GPIHBP1 and LMF1 mRNA in rat tissues, total RNA was extracted from adipose tissues using TRIzol reagent (Life Technologies) and treated with DNA-free kit (Ambion). cDNA was prepared from 50 ng total RNA using Moloney Murine Leukemia Virus Reverse Transcriptase, RNase H Minus (Fermentas) and pd(N)_6_ Random Hexamer (Fermentas) in total volume of 20 μl. The expression of the genes of interest were quantified by real time PCR using TaqMan Universal PCR Master Mix and the ABI Prism 7700 Sequence Detection System (Applied Biosystems, Foster City, CA) and normalized to endogenous control (18S rRNA). ANGPTL4, LPL, GPIHBP1 and LMF1 primers and probes were designed from the published sequences for rat ANGPTLl4, LPL, GPIHBP1 and LMF-1 (corresponding GenBank accession numbers are: NM_199115, NM_000237, NM_001130547, and XM_340769. The sequences for primers and probes used were as follows:

5′-Fam-CTTGGAGCCCATGCTGCTGGC-TAMRA (LPL probe)

5′-ACTGGTGGGACAGGATGTGG (LPL forward primer)

5′-CCGTTCTGCATACTCAAAGTTAGG (LPL reverse primer)

5′-Fam-TCCCCAAGGCGAGTTCTGGCTG- TAMRA (ANGPTL4probe)

5′-GACGCCTGAACGGCTCTGT (ANGPTL4forward primer)

5′-CCCCTGTGATGCTGTGCAT (ANGPTL4reverse primer)

5′-Fam-TGCCAGCACGAAATTCTCCCCG-TAMRA (GPIHBP1 probe)

5′-TCAAAGGCTCATTCTCATCTTGA (GPIHBP1 forward primer)

5′-ACACTCTTGGTTTCCTTCCAACA (GPIHBP1 reverse primer)

5′-Fam CGCTTTCATTTACTTTGTGGCCTTCTTGG-TAMRA (LMF1 probe)

5′-CAGCCTGGCTACACACGGGC (LMF1 forward primer)

5′-CAGCCAGTGCTGCCGTGGAA (LMF1 reverse primer)

Expression levels were normalized to 18S rRNA using the Eukaryotic18S rRNA Endogenous Control Reagent Set supplied from Applied Biosystems. All calculation was done in accordance with recommendation from Applied Biosystems (User Bulletin #2).

For analyses of mRNAs for LPL, GPIHBP1 and ANGPTL4 in mouse adipose tissue, total RNA was isolated with TRIzol reagent (Invitrogen, Breda, The Netherlands) according to manufacturer’s instructions. One μg of total RNA was reverse transcribed using iScript (Bio-Rad, Veenendaal, The Netherlands). cDNA was amplified on a Bio-Rad CFX384 Real Time System using Sensimix (Bioline, GC Biotech, Alphen aan de Rijn, The Netherlands). Cyclophilin was used as housekeeping gene. PCR primer sequences were taken from the PrimerBank and ordered from Eurogentec (Seraing, Belgium). Sequences of the primers used are available upon request.

### Gene expression array

Food was removed from one group of rats (n=6) at 1:30 h while another group (n=6) remained fed *ad lib*. The animals were killed starting from 8:15 h and epididymal adipose tissue was collected. Aliquots of total RNA were converted to biotinylated double-stranded cRNA using Illumina Totalprep RNA Amplification Kit according to manufacturer’s instructions (Ambion, Austin, TX, USA). The labeled cRNA samples were hybridized to RatRef-12 Expression BeadChip (Illumina, San Diego, CA, USA), incubated with streptavidin-Cy3 and scanned on the Illumina Beadstation GX (Illumina, San Diego, CA, USA). To determine differentially expressed genes microarray data were analyzed using Illumina Beadstudio software (version 3.3). The data were normalized and significant differential expression was calculated using Beadstudio’s cubic spline algorithm. False discovery rate was applied. The gene expression fold change for the fasted group was calculated as the average signal value relative to the average signal value for the *ad lib* fed group. Statistical significance cutoff was set to P < 0.01; minimum signal intensity was set at 50 A. Full data are deposited at NCBI GEO, accession no GSE41800.

### Western analyses

The same homogenates as prepared for assay of LPL activity and mass were used. Protein was determined by the BDH protein assay (Pierce) and 20 μg of total protein was separated on 4-20% Tris-glycine gradient gels (Lonza), and blotted onto PVDF membranes (Hybone-C, Amersham Biosciences). The membranes were blocked by ECL Advance blocking agent (GE Healthcare). For detection of LPL we used a rabbit polyclonal antibody from Santa Cruz Biotechnology at a dilution of 1:2000. For detection of ANGPTL4 we used a polyclonal goat anti-human antibody against the N-terminal domain (N-15, Santa Cruz Biotechnology), a goat polyclonal anti-mouse against the C-terminal domain (L-17, Santa Cruz Biotechnology) and a rabbit polyclonal antibody against full-length ANGPTL4 from Abcam, Cambridge, UK. All of these antibodies were used at a dilution of 1:3000. For detection we used the ECL detection system (GE Healthcare).

### Metabolite assessments and plasma lipid profiles

For rats, NEFA-HR (WAKO Chemicals, Germany) was used for measurements of plasma NEFA. Plasma insulin was measured using coated plates from Mercodia, Sweden. Trig/GB (Roche Diagnostics, Germany) was used for TG measurements. One Touch Ultra (Life Scan, Milpitas, CA) glucose sticks were used for blood glucose determinations on whole blood immediately after collection.

For mice, plasma cholesterol was determined using the Cholesterol PAP SL kit from Elitech (Sopachem, Ochten, Netherlands). Plasma triglycerides were determined using the GPO PAP kit from Instruchemie (Delfzijl, Netherlands). Plasma lipoproteins were separated using fast protein liquid chromatography (FPLC). For this 0.2 ml of pooled plasma was injected into a Superose 6B 10/300 column (GE Healthcare Bio-Sciences AB, Roosendaal, Netherlands) and eluted at a constant flow of 0.5 ml/minute with phosphate buffered saline (pH 7.4). The effluent was collected in 0.5 ml fractions and TG and cholesterol levels were determined.

### Statistical analysis

Data are presented as mean ± SEM. Statistical analyses were performed using unpaired *t*-test of variances or one-way ANOVA, where *= p<0.05, ** = p<0.01 and *** = p<0.001. Unless otherwise stated, comparisons were made to time = 0 min or to matched controls.

## Abbreviations

ANGPTL: Angiopoietin-like protein; ActD: ActinomycinD; CHX: Cycloheximide; GPIHBP1: Glycosylphosphatidylinositol-anchored high density lipoprotein-binding protein 1; LPL: Lipoprotein lipase; NEFA: Non-esterified fatty acids also called free fatty acids FFA; PPAR: Peroxisome proliferator activated receptor.

## Authors’ contributions

OK, GO, and TO did the conception and design of the research and conducted most of the animal experiments. OK performed most of the analytical work. VS, GO, and TO had conducted preliminary experiments on which the study was designed. EV and SKN contributed to mRNA analyses and to design and performance of the gene array study, respectively, together with OK. RS, FM, and SK conducted the experiments on *Angptl−/−* mice and most of the analyses on these animals. All authors contributed to analyses of data and to interpretation of the results. OK, GO and TO prepared the figures. GO and TO drafted the manuscript. All authors contributed to edition and revision of the manuscript. KS, GO, and TO approved the final version.
